# LEyes: A lightweight framework for deep learning-based eye tracking using synthetic eye images

**DOI:** 10.3758/s13428-025-02645-y

**Published:** 2025-03-31

**Authors:** Sean Anthony Byrne, Virmarie Maquiling, Marcus Nyström, Enkelejda Kasneci, Diederick C. Niehorster

**Affiliations:** 1 MoMiLab, IMT School for Advanced Studies Lucca, Lucca, Italy; 2 Human-Centered Technologies for Learning, Technical University of Munich, Munich, Germany; 3 Lund University Humanities Lab, Lund University, Lund, Sweden; 4 Department of Psychology, Lund University, Lund, Sweden

## Abstract

Deep learning methods have significantly advanced the field of gaze estimation, yet the development of these algorithms is often hindered by a lack of appropriate publicly accessible training datasets. Moreover, models trained on the few available datasets often fail to generalize to new datasets due to both discrepancies in hardware and biological diversity among subjects. To mitigate these challenges, the research community has frequently turned to synthetic datasets, although this approach also has drawbacks, such as the computational resource and labor-intensive nature of creating photorealistic representations of eye images to be used as training data. In response, we introduce “Light Eyes” (LEyes), a novel framework that diverges from traditional photorealistic methods by utilizing simple synthetic image generators to train neural networks for detecting key image features like pupils and corneal reflections, diverging from traditional photorealistic approaches. LEyes facilitates the generation of synthetic data on the fly that is adaptable to any recording device and enhances the efficiency of training neural networks for a wide range of gaze-estimation tasks. Presented evaluations show that LEyes, in many cases, outperforms existing methods in accurately identifying and localizing pupils and corneal reflections across diverse datasets. Additionally, models trained using LEyes data outperform standard eye trackers while employing more cost-effective hardware, offering a promising avenue to overcome the current limitations in gaze estimation technology.

## Introduction

Gaze estimation refers to the computational techniques employed to estimate an individual’s gaze location. Commonly, algorithms in this field use eye images as inputs and yield an estimated gaze point or gaze direction (Akinyelu & Blignaut, 2020). This field of research has recently witnessed a surge of new interest driven by technological advancements across various domains. Most notably, the widespread adoption of Virtual Reality (VR) headsets (Byrne et al., 2024; Garbin et al., 2020; Palmero et al., 2021), the integration of eye-tracking technology into smartphones and tablets (Krafka et al., 2016; Valliappan et al., 2020), and the continuous improvement of both wearable eye-tracking devices (Santini et al., 2019) and augmented reality systems (Renner & Pfeiffer, 2017) have fueled this growing interest. More generally, eye tracking sees application across a wide array of fields, such as healthcare (Pierce et al., 2016), economics (Lahey & Oxley, 2016; Byrne et al., 2023), neuroscience and cognitive sciences (Hessels & Hooge, 2019; Niehorster et al., 2019), and education (Strohmaier et al., 2020; Jarodzka et al., 2017). Eye tracking also has applications in human-computer interaction, such as enabling foveated rendering (Mohanto et al., 2022; Walton et al., 2021), realistic eye contact between avatars (Yassien et al., 2020) and assisting users with mobility impairments (Meena et al., 2017). Finally, eye tracking serves as a promising tool for technical training and evaluation in fields such as surgery (Chetwood et al., 2012; Tien et al., 2014), dentistry (Castner et al., 2020), and aviation (Niehorster et al., 2020).

A prevalent gaze estimation technique involves video observation of the eyes of a participant. To determine the gaze direction or eye orientation from these videos, often the position of both the pupil (P) and one or multiple corneal reflections (CRs) are used. The quality of the data provided by this process, known as P-CR eye tracking (Merchant et al., 1974; Hansen & Ji, 2010), depends on the accuracy with which the pupil and the CRs are localized. As such, much research effort has been spent on accurately and robustly localizing the pupil and CR features in eye images (e.g., Santini et al., 2018a, b; Nyström et al., 2023; Sadeghi et al., 2024).

Deep learning methods have significantly enhanced both the accuracy and robustness of gaze estimation techniques, as evidenced by multiple studies (e.g., Fuhl et al., 2016, 2017; 2023; Kim et al., 2019; Kothari et al., 2021; 2022; Maquiling et al., 2023; Nair et al., 2020), especially in noisy images. Deep learning algorithms address issues present in conventional algorithmic approaches, which are vulnerable to unpredictable factors like blinks or reflections in the recording (Kothari et al., 2022). Yet despite these benefits, the practical implementation of deep learning algorithms continues to pose challenges, principally due to the complex tasks of finding suitable training data (Garbin et al., 2020; Palmero et al., 2021). This data procurement obstacle in gaze estimation can be detailed as follows:

1.  *Data scarcity:* While data scarcity is a common issue across many deep learning domains (Bansal et al., 2022), this challenge is particularly acute in the field of eye-tracking research. Collecting a sufficient amount of training data for the development of deep learning models in this area demands significant time and resources (Byrne et al., 2023; Garbin et al., 2020).
2.  *Annotation of datasets:* The second challenge involves the necessity for annotating segmented regions within eye images. This annotation is essential for creating labels for supervised learning algorithms that train deep learning models. It is a process that is not only time-consuming but also often requires extensive expertise in the domain when providing the manual labels (Garbin et al., 2020; Palmero et al., 2021).
3.  *Differences between available datasets:* The third challenge compounds the issue of data scarcity and stems from disparities in eye images found in the limited amount of publicly available datasets. Differences can occur not just across recording setups but also from variation in eye image attributes like iris brightness, which lead to pixel-level differences that contribute to sub-optimal network performance (Nair et al., 2020). This is a major issue as slight differences can have a substantial impact on model performance and generalizability.
4.  *Computational resources:* In instances where appropriate training datasets are obtainable, the computational demands associated with model training frequently exceed the resources accessible to academic researchers, particularly those affiliated with less well-endowed institutions.

A proposed solution to the first three challenges is the use of synthetic datasets, which allows for the generation of vast amounts of annotated images (Kim et al., 2019; Byrne et al., 2023; Maquiling et al., 2023; Nair et al., 2020). Synthetic data has been used successfully to train deep neural networks in fields such as medical imaging (Gao et al., 2023), autonomous driving (Osiński et al., 2020), and microscopy (Helgadottir et al., 2019). The efficiency of training using synthetic data varies significantly with the chosen method for generating the dataset, and depends on at least two factors: storage requirements and the level of photorealism of the synthetic data. Some methods require that the entire dataset needs to be pregenerated before training the model, which entails significant storage requirements.

For instance, the creation of the NVGaze dataset took 30 s per image, cumulatively requiring approximately 3.8 years on a single GPU and multiple Terabytes of storage—this duration was notably reduced to a week with access to a supercomputer (Kim et al., 2019). Other methods can, however, train neural networks with synthetic images that are generated on the fly (Helgadottir et al., 2019; Byrne et al., 2023; Maquiling et al., 2023; Midtvedt et al., 2021). Such methods, especially when they use computationally inexpensive synthetic data pipelines for generating the images, allow for the training of models in a very resource-efficient manner.

Typically in the field of gaze estimation, synthetic eye image creation methods aim for photorealism by employing a 3D model of the human eye and surrounding facial region to produce images that are as similar as possible to those captured by eye-trackers, using render software or game engines such as Blender or Unity. The goal of these methods is to match the synthetic dataset’s underlying distribution with the variability seen in datasets of recorded real-world eye images (Wood et al., 2015; Nair et al., 2020). The photorealistic synthetic data approach, however, is not without limitations. One key challenge is the complexity of generating synthetic datasets that accurately capture the distribution of real eye images. Additionally, concerns exist regarding the potential for achieving state-of-the-art outcomes when compared to models trained on genuine eye images (Nguyen et al., 2024). For example, one study illustrated a decline in gaze estimation accuracy by 1° when comparing a model trained on photorealistic synthetic images to one trained on a subset of real eye images using a neural network (Kim et al., 2019; Nair et al., 2020). This may be because numerous intricate features must be precisely constructed during synthetic dataset creation, and even minor deviations in design can significantly impact a model’s inference capabilities during gaze estimation. We argue that the pursuit of photorealism is both unnecessary to produce accurate results and resource-intensive in terms of both dataset construction and model training.

Here, we introduce LEyes, a novel approach to training gaze estimation models. The key hypothesis behind our approach is that, in the end, a model that localizes or segments eye features such as pupils or CRs is nothing more than a model that is good at finding or delineating relatively dark or light pupil- or CR-shaped blobs in an image. It may be expected that such models can be effectively trained on any set of training images with feature and image intensity distributions that encompass what is encountered in real eye images, regardless of whether these images look like eye images to a human observer. Releasing the constraint of the photorealistic approach that training images should look as similarly as possible to real eye images allows us to use light-weight procedures for generating training images on the fly. This obviates much of the storage and computational resource needs of the photorealistic approach to training models for gaze estimation, which has several benefits. Firstly, the method requires no special computational resources and can be trained on online platforms such as Google Colab, which are accessible to everyone and can often be run free of charge. This enables also researchers without access to dedicated deep-learning hardware facilities to employ and develop new gaze estimation technology, democratizing access. Secondly, due to the low computational demands, our approach could serve as a quick but effective pre-training step, or could be employed for on-device adaptation to a specific user.

The potential of this approach has been previously explored for a very simple scenario involving just localizing the center of a single CR (Byrne et al., 2023) and in preliminary work for identifying multiple CRs (Maquiling et al., 2023). This study evaluates the suitability of the LEyes method for a range of tasks involving both pupils and CRs, enabling the creation of complete gaze estimation pipelines. Our focus in this study is not on model innovation with the aim of beating state-of-the-art eye image segmentation or eye feature localization performance but on showing the potential of the LEyes method to train neural networks for such tasks. As such, we will only employ standard neural networks architectures with parameter counts comparable to other models (such as Chugh et al., 2021; Kothari et al., 2021; Niu et al., 2021) in this paper, so that good performance of our method can likely be ascribed to appropriate training data and not to use of a very powerful model. To evaluate our method, several neural networks are constructed for different estimation tasks including localizing and segmenting eye features, but also the more complex task of detecting multiple CRs and simultaneously identifying which CRs correspond to which illuminators. Using LEyes, each network is trained on simplified images that abstractly represent the features in eye images that are relevant for the given estimation task, rather than on images mimicking real eyes. The results show that our approach performs on par with, and often outperforms, other synthetic data approaches and standard computer vision methods in accuracy across diverse eye tracker setups.

**Fig. 1:**
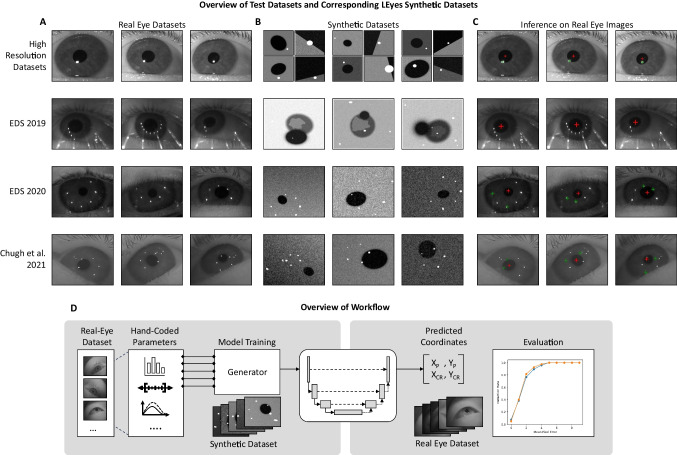
**A.** Images from the datasets we used to test the LEyes framework. **B.** The LEyes synthetic training sets corresponding to the real eye datasets in A. These images are based on the light distributions of the real eye datasets. **C.** The predictions of the LEyes trained models on the real eye images. **D.** An overview of our approach: First, we establish a set of parameters based on the distributions of the collected data. These distributions pertain to pixel-level details like the iris and pupil intensity. Next, we employ a generator to efficiently produce new synthetic images from these parameters on the fly. The generated images are used to train a neural network (such as the depicted U-Net), which is then tested on real eye images recorded from the same device

## Methods

### Overview of the LEyes framework

As introduced, the key difference between the LEyes framework and previous photorealistic approaches to training neural networks on synthetic eye images is that LEyes images are not designed to look like real eyes (see Fig. 1B, for example LEyes images). Releasing the constraint that training images should look “real” not only allows for much simpler images that can be generated swiftly, but also allows for training models on images with larger distributions of parameters (e.g., feature shape and luminance distributions) than would be encountered in real images, potentially increasing robustness of the resulting model to variations in the input images.

LEyes images are created employing the generator function from the DeepTrack 2.1 package (Midtvedt et al., 2021). This generator function enables the creation of training images from a set of feature parameters describing the image swiftly and on-the-fly, which contrasts favorably with the resource-intensive photorealistic approaches discussed above. DeepTrack’s generator function also manages image-label pairings during training, avoiding the need for pre-generated images, thereby conserving disk space (Helgadottir et al., 2019). A final benefit of using the DeepTrack generator for on-the-fly image generation is that it allows us to create a new batch of images before each training step. This entails that each image is shown to the network only once and then discarded, which permits very efficient use of the computer memory and, more importantly, helps prevent overfitting (compared to training regimes that reuse data) and avoids the need for real-time validation of network performance. Comprehensive details on our methodologies, synthetic data pipelines, and the model training regimes, along with the trained models and evaluation code, are available at our GitHub repository: https://github.com/dcnieho/Byrneetal_LEyes.

How are LEyes images designed? While we posit that effective gaze estimation models can be trained on images that do not resemble real eyes, it is crucial that the training images contain the necessary information for the model to learn and perform the task it is posed. Photorealistic approaches tackle this problem by capturing key physical aspects of the eye and eye tracker, such as lighting setups, camera parameters, and the material properties of various eye structures, in their simulation to replicate real-world conditions accurately (Kim et al., 2019; Nair et al., 2020; Wood et al., 2015). Such synthetic images go beyond mere visual mimicry; they deeply simulate the interaction between light and the camera sensor, capturing the essence of how features are recorded in real scenarios.

For LEyes images, we took a different approach. There are two aspects to modeling the input for a given task that a neural network would have to solve: (1) what features to include; and (2) what parameter distributions to use for them. Essentially this is asking what information should be put in the synthetic images and how should the variability in this information be represented between images so that the training distribution is sufficiently wide to encompass the distribution of real eye images the neural network will encounter.

Like the training of deep learning models in general (e.g., deciding hyperparameters), deciding on the parameters for generating the synthetic images is not an exact science and may involve some trial and error. Our general approach to modeling features such as the pupil, iris, and CRs effectively was to combine knowledge about the physics of the problem (e.g., the appropriate shape of features and relative luminance relations between features) with luminance attributes derived from distributions of data recorded across various eye-tracking setups. To determine suitable luminance distributions for pupils and their backgrounds, we analyzed the dataset for which the model is intended by extracting the mean luminance and standard deviation of the pupil region and the surrounding area (part of the iris). The resulting empirical luminance distributions were used as guidance for setting the pupil and background luminance parameters in the LEyes synthetic image pipeline. Specifically, these parameters were set to exceed the observed luminance ranges. This approach accounted for the limited participant pool, which might not capture the extreme values that recording devices are capable of producing. For other parameters, such as pupil shape, position, etc, we used distributions that are significantly wider than those that would be observed in real eye images. This design reflects the goal of LEyes to simulate the range of possible appearance of eye-tracking features in the images a camera might capture, rather than reproducing data specific to the participants in the dataset, thereby enhancing generalizability and ensuring robust model performance across diverse recording conditions.

Decisions about which image features to include in a synthetic image generation pipeline were guided by the principle of simplicity: features were only included if they were expected to enhance robust model performance. An example of this is the decision to include a luminance gradient for the background in the synthetic data pipelines for the Chugh et al. (2021) and OpenEDS2020 datasets. This adjustment addressed issues where dark scleral regions in the dataset were occasionally misclassified as pupils. Table 1 provides an overview of the parameters of the synthetic data pipelines used in this paper. These synthetic image sets are designed to offer straightforward yet realistic representations of eye features, capturing sufficient variability to support model generalizability across diverse recording conditions and datasets.

LEyes demonstrates that such images can encapsulate eye features that are relevant for eye tracking, and that by means of such abstract images one can efficiently embody both the eye characteristics and aspects of the physical eye tracker setup for neural network training. Below we discuss, in order, the datasets that were used for testing the LEyes framework, the variety of employed deep learning architectures and evaluated estimation tasks, the training regimes employed, the pipelines used to generate synthetic training data for each of the data sets, and finally further image pre-processing and model output post-processing and data analysis procedures.

### Datasets

To evaluate the effectiveness of LEyes in a broad range of settings, we utilized both two high-resolution eye image datasets collected in a lab setting, and three widely recognized datasets collected from eye trackers embedded in virtual reality (VR) headsets. The broad range of datasets allowed us to evaluate the applicability and performance of LEyes across different eye-tracking technologies and for a range of different estimation tasks. Example images for each dataset are shown in Fig. 1A.

**Table 1:** Overview of the parameters of the synthetic data pipelines in this paper

Parameter	Explanation	Model(s)	Examples
Feature parameters			
$x_c$	Horizontal location of feature center	All	
$y_c$	Vertical location of feature center	All	
$\sigma _\alpha$	Spread along the minor axis of the 2D Gaussian	All	
$\sigma _\beta$	Spread along the major axis of the 2D Gaussian	All	
$\theta$	Orientation of the 2D Gaussian	All	
*A*	Amplitude of the 2D Gaussian	All	
Luminance parameters			
$L_{CR}$	Luminance of a CR	All	
$L_p$	Luminance of a pupil	All	
$L_{col}$	Luminance of the collarette	EDS2019	
$L_i$	Luminance of the iris	All	
$L_s$	Luminance of the sclera	EDS2019	
CR constellation parameters			
*w*	Width of the polygon	Chugh et al. (2021)	
$f_b$	Width reduction factor of the base of the polygon	Chugh et al. (2021)	
*h*	Height of the polygon’s base	Chugh et al. (2021)	
$h_r$	Height of the roof above the polygon’s base	Chugh et al. (2021)	
$\phi$	Orientation of the polygon	Chugh et al. (2021); EDS2020	
*m*	Radius of the polygon	EDS2020	
Other parameters			
$\sigma _n^{2}$	Standard deviation of pixel noise	All	
$N_{CR}$	Number of CRs	Pupil; EDS2019	
$N_{spur}$	Number of spurious reflections	EDS2019; Chugh et al. (2021); EDS2020	
$N_{vert_{col}}$	Number of collarette vertices	EDS2019	
$r_{col}$	Radius of collarette	EDS2019	
*p*	Feature drop-out rate	Chugh et al. (2021); EDS2020	

The “Model(s)” column indicates in which models the parameter was used. For clarity, some parameter manipulations are more extreme than used in the synthetic data pipelines in this paper

#### High-resolution eye-tracking datasets

**Fig. 2:**
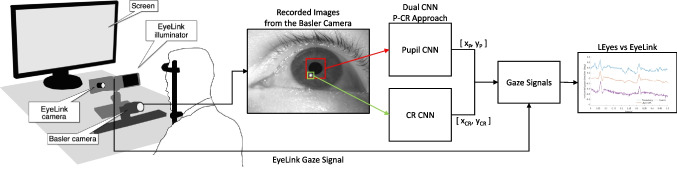
Experimental setup: In a co-recorded setup, we acquire eye images from the FLEX setup and gaze signals from the EyeLink 1000 Plus. We analyzed the eye images we recorded from expert participants using a dual-CNN approach. The pupil CNN localized the pupil center, while the CR CNN localized the center of the CR located in the eye image. Both CNNs achieved sub-pixel localization error. Image of co-recording setup adapted from (Nyström et al., 2023)

To evaluate whether the LEyes framework is suitable for use in a high-end, high-resolution eye-tracking setting, we collected data using a laboratory eye-tracking setup. High-resolution eye images were recorded from the first, third, and last author of the current paper and one further experienced participant with the FLEX setup (Nyström et al., 2023; Hooge et al., 2021; Valtakari et al., 2024). Eye movement data were simultaneously recorded with the EyeLink 1000 Plus (SR Research Ltd., Ottawa, Canada). Such co-recording was required since the eye images captured by the EyeLink are not accessible and a direct comparison of its image processing to the LEyes method is thus not possible. The setup is shown in Fig. 2. The EyeLink illuminator was used to illuminate the eye and create the corneal reflection used by both the EyeLink and the FLEX setup. The FLEX setup used a Basler ace acA2500-60um camera equipped with a 50-mm lens (AZURE-5022ML12M) and a near-IR long-pass filter (MIDOPT LP715-37.5) that was positioned 50 cm from the participant’s eyes.

To provide additional variation in the luminance profiles of the eye images and thereby test the robustness of our model, two different datasets were collected using the same four participants: the FLEX (1) acquired images at 1000 Hz and (2) acquired images at 500 Hz. These datasets will be referred to as the 1000-Hz and 500-Hz datasets, respectively. For both datasets, the left eye was filmed. Camera and illuminator settings for the two data sets were as follows:

1. *1000 Hz*. 8-bit images were captured at 672 x 340 pixels, with camera exposure set to 882µs and gain to 12 dB. EyeLink illuminator power was 100%.
2. *500 Hz*. 8-bit images were captured at 896 x 600 pixels, with camera exposure set to 1876µs and gain to 10 dB. EyeLink illuminator power was 75%.

It should be noted that the same optics and recording distance were used for both datasets, and that the lower resolution of the 1000-Hz dataset was only due to a tighter cropping of the image sensor data that was required to achieve the higher frame rate. As such, the sizes of features (pupil and CR) and the distances between them are directly comparable between the two datasets. The captured eye images were brighter at the 500-Hz than the 1000-Hz sampling rate due to the longer possible exposure time. Videos were captured with custom software that streamed the recorded frames to mp4 files using libavcodec (FFMpeg) version 5.1.1 and the libx264 h.264 encoder it includes (preset: veryfast, crf: 17, pixel format: gray).

For both datasets, simultaneous binocular eye movement recordings were performed at 1000 Hz with an EyeLink 1000 Plus (host software 5.12) in desktop setup using the center-of-mass pupil tracking mode. The EyeLink camera sensor was located 56 cm away from the participant’s eyes. To synchronize the acquisition of eye images from the FLEX with eye movement data from the EyeLink, TTL triggers were sent to the EyeLink Host computer at the onset and offset of each FLEX image recording trial. The recordings took place in a dark room with no windows.

Several tasks were shown on an Asus VG248QE monitor at 60 Hz (viewing distance 79 cm). Participants performed the following tasks while stabilized on a chin- and forehead rest:

1. Nine 1-s fixations in random order on a $3\times 3$ grid of fixation points positioned at $h = \{-7, 0, 7\}$ deg and $v = \{-5, 0, 5\}$ deg.
2. One 30-s fixation on a point positioned at $h = 0$ deg and $v = 0$ deg while the background luminance alternated between black and white at a cycle time of 3s.
3. A total of six 30-s fixations on points positioned at $h = \{-3.5, 0, 3.5\}$ deg and $v = 0$ deg on a middle grey background, with each position repeated twice.
4. Five rightward step-ramp pursuit trials where a dot moved from $h=-10$ deg to $h=10$ deg at a speed of 2°/s following a 200ms leftward step.
5. A 30-s sequence of fixations on a dot that was presented for 1 s at positions $(x, 0), x \in \{-7,-3.5,0,3.5,7\}$ deg, with each position repeated six times.
6. Ninety fixations in random order on a dot that was presented for 1.5 s at positions $h = \{-7,-3.5,0,3.5,7\}$ deg and $v = \{-5,0,5\}$ deg, with each position repeated six times.

The fixation point consisted of a blue disk (1.2° diameter) with a red point (0.2° diameter) placed on its center. These datasets have previously been used in Byrne et al. (2023).

The reader may note that the gaze angles in this dataset only covered ± 7° horizontally, and ± 5° vertically. This range of gaze angles was chosen because we consider it representative of the gaze angles used in many high-resolution eye-tracking settings, and since we have no reason to expect that our results would not be representative of those expected when using larger gaze angles, as long as the CR does not roll off the cornea (Holmqvist et al., 2011). It should furthermore be noted that the precision of traditional high-resolution eye trackers is usually highest under small gaze angles (Holmqvist & Andersson, 2017; Nyström et al., 2013). As such, using small gaze angles in our evaluation means that the traditional methods could be expected to perform well, providing a hard test for the LEyes method.

The total recording time for each participant was approximately 8.5 min, resulting in a database containing approximately 437,500 FLEX eye images per participant at 1000 Hz and 219,300 images at 500 Hz, along with the EyeLink data.

#### VR datasets

The OpenEDS 2019 dataset, described in Garbin et al. (2020), originates from a VR head-mounted display with dual eye-facing cameras, capturing images at 200 Hz in controlled lighting. It comprises eye-region videos from 152 participants, yielding 12,759 images with pixel-level segmentation annotations for the iris, pupil, and sclera, based on human-annotated key points.

Next, we used the OpenEDS 2020 Challenge dataset (OpenEDS 2020, Palmero et al., 2021), which includes data from 200 participants and features manually annotated segmentation labels for the pupil in 5% of the data, resulting in 2,605 images. These images were captured at 100 Hz using a VR headset under controlled lighting conditions.

Additionally, we employ a dataset by Chugh et al. (2021), who captured eye images from 15 participants through a VR headset with an eye-tracking attachment. This dataset is distinguished by its manual annotations of both the pupil center and the centers of up to five corneal reflections (CRs), and information about which CR corresponded to which of the five illuminators in their setup.

Each of these datasets, derived from separate recording devices and conditions, serves as a distinct evaluation platform for LEyes, allowing us to gauge its performance across varied hardware setups. For detailed information about these VR datasets, readers are directed to the original publications. Evaluation using LEyes was run on the entirety of each dataset, not only on the subset marked as “testing set” by the dataset authors.

### Deep leaning architectures

To highlight the adaptability of the LEyes framework, we engineered an array of neural networks, each grounded in established architectures or building upon prior research (Byrne et al., 2023; Helgadottir et al., 2019; Niu et al., 2021; Maquiling et al., 2023). Among these, we designed a dual convolutional neural network (CNN) approach for PC-R eye tracking on the images in the high-resolution dataset. This setup involves one CNN dedicated to pinpointing the pupil center and another for identifying the center of the corneal reflection (CR), as depicted in Fig. 2. For inference on the VR datasets, we opted for various iterations of the popular U-Net model (Ronneberger et al., 2015), each specifically adjusted and trained on images generated by LEyes.

#### Dual CNN approach for high-resolution datasets

A CNN model was trained to localize the subpixel center of an eye feature separately for the pupil and CR. Overall, four CNNs were trained: two for localizing the pupil and CR centers in data captured at 500 Hz with the FLEX setup and another two for data captured at 1000 Hz. Each model is composed of seven convolutional layers followed by two dense layers. The CNNs for CR center localization in both 500-Hz and 1000-Hz data, as well as the CNN trained to detect the pupil center in 500-Hz data, have the following convolution layer dimensions: (64, 64, 128, 128, 256, 256, 512) while the pupil CNN for 1000-Hz data has wider dimensions: (128, 128, 256, 256, 512, 512, 768). The CR CNNs have dense layers with sizes of (64, 32) while the pupil CNNs both have sizes of (64, 64). The CR CNNs both have a total of 6,268,386 trainable parameters while the pupil CNN for 500-Hz and 1000-Hz data have 6,270,530 and 19,671,426 trainable parameters, respectively. Each CNN model was built within the DeepTrack 2.1 library (Midtvedt et al., 2021).

#### U-Net models for VR datasets

In the case of the EDS 2019 dataset, we adopted a standard U-Net model (Ronneberger et al., 2015) from the PyTorch Segmentation Modules library (Iakubovskii, 2019). This model has a ResNet-34 backbone pre-trained on the ImageNet dataset (Deng et al., 2009; Koonce & Koonce, 2021) within its encoder, and its decoder is composed of five convolutional layers with the following dimensions: 256, 128, 64, 32, and 16. The U-Net is designed to process grayscale images of any size, outputting a probability map for pupil segmentation. The total number of trainable parameters in this U-Net configuration is approximately 24.43 million.

For the EDS 2020 and the dataset by Chugh et al. (2021), a customized U-Net model was utilized, drawing from methodologies established in earlier research (Niu et al., 2021). This model features an architecture with six residual modules within both the encoder and decoder, maintaining a consistent channel size of 256 but altering the spatial dimensions through strategic down- and up-sampling. The model’s output is directed through two convolutional blocks, tasked with generating heat maps for the CRs and the pupil.

### Model training regimes

This section outlines the procedures and parameters used for training the models. Consistent with common practices in deep learning, several parameters, such as batch size, loss function, and optimizer, were determined iteratively based on model convergence observed during training.

#### CNNs for high-resolution datasets

Following our previous work (Byrne et al., 2023; Maquiling et al., 2023), we adopted a two-stage approach for training each CNN, training first on synthetic images with harder examples and then honing in on cases that are closer to the dataset. The generator was configured to present the model with 1000 unique samples per epoch, with batch sizes of four for the CR CNNs, 16 for the pupil CNN at 500 Hz, and eight for the pupil-CNN at 1000 Hz. The batch size was further reduced to four for both pupil CNNs during the second stage of training. Additionally, a set of pre-generated synthetic images was used for validation, with a validation set size of 300 for the CR CNNs and 600 for the pupil CNNs. We employed the mean squared error (MSE) loss function for the CR CNNs and the mean absolute error (MAE) loss function for the pupil CNNs, and to assess model performance. To train the models, we used the Adam (Kingma & Ba, 2014) optimizer for the CR CNNs and the pupil CNN at 1000 Hz, while AdamW was used for the pupil CNN at 500 Hz. In the first stage, the initial learning rate was set to $1e^{-4}$, which was subsequently decreased to $1e^{-6}$ in the second stage. An exponential decay scheduler was used for the learning rate in all training regimes.

The CR CNNs at 500 Hz and 1000 Hz were trained for a maximum of 700 epochs for the first and second stages, incorporating an early stopping mechanism to prevent overfitting. The first stage of the CR CNN at 500 Hz converged after 286 epochs while the second stage required 555 epochs. The 1000-Hz model reached convergence in 167 epochs for the first stage and 307 epochs for the second stage.

In the first stage, the pupil CNNs are allowed to train up to 500 epochs with a patience of 20. In the second stage, the 500-Hz model is trained for up to 40 epochs with a patience of 5 while the 1000-Hz model is trained for up to 100 epochs with a patience of 10. The 500-Hz model reached convergence after 99 epochs in the first stage and 25 epochs in the second stage. The 1000-Hz model achieved convergence after 88 epochs in the first stage and 36 epochs in the second stage.

In the second stage, the first convolutional layer of each model is frozen and we used an iterative approach to determine which additional layers to freeze, if any. We chose to freeze the first convolutional layer for the pupil CNNs and the 1000-Hz CR CNN, and the first two layers of the CR CNN at 500 Hz.

#### U-Net models for VR datasets

To train the U-Net for the OpenEDS 2019 dataset, we chose the AdamW (Loshchilov & Hutter, 2019) optimizer with an initial learning rate set to $1e^{-4}$ and an exponential decay scheduler. The loss used is a combination of Binary Cross Entropy loss (Ruby & Yendapalli, 2020), Dice loss (Li et al., 2020), and Focal loss (Lin et al., 2017). During the training phase, the model was shown 1000 new synthetic images per epoch and the validation set consisted of 400 pre-generated synthetic images. The model training ran for 100 epochs reaching a natural plateau.

Similar to the above, the U-Net model used for Chugh’s dataset (Chugh et al., 2021) is trained in two stages. The first stage consisted of a broader range of challenging examples, aimed at enhancing the model’s robustness to large variations in eye data while the second stage consisted of images that more closely represent the images captured by the eye tracker. Similar to the U-Net model for EDS 2019, we used the AdamW optimizer with an initial learning rate of $1e^{-4}$ in the first stage and $1e^{-5}$ in the second stage, an exponential decay scheduler, and a combination of Binary Cross Entropy loss (Ruby & Yendapalli, 2020), Dice loss (Li et al., 2020), and Focal loss (Lin et al., 2017) for the loss. The generator was first configured to present the model with 20000 unique images per epoch. In the second stage, the generator is reconfigured to show 1000 images. We let the model train for 30 epochs in the first stage and 20 epochs in the second stage. We incorporated early stopping with a patience of 30 for the first stage and 5 for the second stage.

Similarly, the U-Net model for EDS 2020 (Palmero et al., 2021) is trained in two stages. We let the model train for 500 epochs in both stages and incorporated early stopping with a patience of 30. In the first stage, a weight of 100 is added to the Binary Cross Entropy Loss, while the rest of the parameters for both the first and second stages remain the same as the EDS2020 U-Net model. In both stages, the generator was configured to produce 1000 unique images per epoch, training stopped early after 175 epochs in the first stage and 81 epochs in the second stage.

### Generating synthetic data for model training

To evaluate our method, we apply our approach to distinct tasks. As detailed above, we have created models for each of these tasks and corresponding datasets. To train these models, we have created specialized synthetic data pipelines for each task, that replicate the light distributions observed in eye images corresponding to the datasets utilized in each task. The synthetic data pipelines we developed use simulations to model the light distributions of the relevant aspects of an eye image that the given model would have to deal with during inference. Here, we first discuss the common elements across the five synthetic data pipelines, followed by an in-depth discussion of each specific pipeline in the subsections. Details on the distinct features of each dataset are provided in the relevant subsection. An overview of the various features in these generated images along with their parameters, the meaning of these parameters and visual examples is given in Table 1.

Previous work (Byrne et al., 2023; Nyström et al., 2023; Maquiling et al., 2023) has illustrated that some of the features in eye images that are essential for eye-tracking, like the pupil and corneal reflections, can be effectively modeled with 2D Gaussian distributions. Here we adopt the same approach and model blob-like features, such as the pupil and CRs, as 2D Gaussian distributions using the equation:

1 $$\begin{aligned} G(x,y) = Ae^{-a(x - x_c)^2 - b(x - x_c)(y - y_c) - c(y - y_c)^2 }, \end{aligned}$$

where

2 $$\begin{aligned} a&= \frac{\cos (\theta )^2}{2\sigma _\alpha ^2} + \frac{\sin (\theta )^2}{2\sigma _\beta ^2},\end{aligned}$$

3 $$\begin{aligned} b&= \frac{\sin (2\theta )}{4\sigma _\alpha ^2} - \frac{\sin (2\theta )}{4\sigma _\beta ^2},\end{aligned}$$

4 $$\begin{aligned} c&= \frac{\sin (\theta )^2}{2\sigma _\alpha ^2} + \frac{\cos (\theta )^2}{2\sigma _\beta ^2}, \end{aligned}$$

and where $\theta$ is the orientation of the 2D Gaussian centered at $(x_c,y_c)$ and $\sigma _\alpha$ and $\sigma _\beta$ its spread along the minor and major axes, respectively.

The luminance of CRs was always set to full white while the luminance of the pupil, if the synthetic data pipeline contained one, was determined per target data set by analyzing the dataset’s eye images on which inference would be run. Regardless of the Gaussian amplitude *A* of the feature, which was varied to create differently steep edges of a feature, the minor and major axis radii of the luminance plateau in each feature (the bright part of a CR, or the dark part of a pupil) were kept constant by parameterizing

5 $$\begin{aligned} \sigma _r = r/\sqrt{-2\log \frac{1}{A}}, r \in \{\alpha ,\beta \}. \end{aligned}$$

To create the final synthetic image, first the relevant features were layered onto a background luminance distribution that differed between the synthetic data pipelines. These layers were then collapsed into a single image by subtracting dark features from the background, and by adding features to the collapsed image of the preceding layers using the operation *max*(*image*, *background*). Pixel noise was added to the final image by adding a value from a Gaussian distribution $X \sim \mathcal {N}(0,\,\sigma _n^{2})$ to the image that was drawn independently for each pixel. Finally, the resulting image was limited to the range [0, 255], scaled to the range [0, 1] and discretized to 256 levels, corresponding to 8-bit camera images. Example synthetic images used for model training for each dataset are shown in Fig. 1B.

#### Synthetic data pipelines for the high-resolution datasets

The CNN for CR center localization used for the 500-Hz data was the same as presented in previous work (Byrne et al., 2023). As such, only the key points of this synthetic data pipeline are described. Circular CRs ($\sigma _\alpha =\sigma _\beta \in [1,30]$, $A\in [2,20000]$) with luminance $L_{CR}=255$ were placed on a background that was made up of two parts, divided by a randomly oriented straight line representing the pupil-iris border that passed close to the CR. On one side of the line the background was dark, with a luminance $L_p$ drawn from an exponential distribution with its scale parameter set to 10 pixel intensity values, and offset 1. The other part of the background was middle grey (pixel intensity value $L_i=128$). The standard deviation of image noise was varied per generated image, with $\sigma _n \in [0,30]$. The synthetic data pipeline used for training the CNN for determining CR centers in the 1000-Hz eye videos were identical to those used for the 500-Hz data, except that the middle-grey part of the background varied in luminance between $L_i \in [32,153]$. For both the 500-Hz and the 1000-Hz models, during the second training stage, the location of the CR center was constrained to a range spanning 1.5 pixels around the image center.

The synthetic data pipelines used for training the CNN for locating pupil centers differed from the pipelines for the CR CNNs in a few ways. First, the generated synthetic images contained a 2D Gaussian representing the darker pupil. Second, the images contained one or multiple bright 2D Gaussians representing CRs that were randomly positioned and could thus overlap the pupil. Third, instead of a background consisting of dark and grey segments separated by a straight line, the background now consisted of a uniform field at a range of grey levels, representing the iris at various illumination levels.

Specifically, a randomly oriented dark 2D Gaussian with minor axis radius $\alpha _p \in [20,60]$ pixels, major axis radius $\beta _p \in [1\alpha _p,1.3\alpha _p]$ and amplitude $A_p \in [2,20000]$ was used to represent the pupil. Its luminance $L_p$ was drawn from an exponential distribution with a scale parameter of 10, and offset 1.

Between one and four corneal reflections ($N_{CR}$) were generated with minor axis radius $\alpha _c \in [4,12]$ and major axis radius $\beta _c \in [1\alpha _c,1.1\alpha _c]$ and $A_c \in [2,20000]$ and randomly positioned. Overlap between CRs was avoided by removing CRs whose center location was closer to another CR than 1.25 times the sum of the major axis radii of the two CRs, and replacing it with a new randomly positioned CR. The background luminance level representing the iris was $L_i \in [64,179]$ pixel intensity values. The standard deviation of image noise was varied per generated image, with $\sigma _n \in [0,30]$.

The synthetic data pipeline used for training the CNN for determining pupil centers in the 1000-Hz eye videos were identical to those used for the 500-Hz data, except that the background luminance level representing the iris was $L_i \in [32,153]$ pixel intensity values to encompass the iris luminance values in the darker 1000-Hz eye images.

For both the 500-Hz and the 1000-Hz models, for the second training stage the location of the pupil center was constrained to a range spanning 1.5 pixels around the image center and only one randomly positioned CR ($N_{CR}$) was generated.

#### Synthetic data pipeline for OpenEDS 2019

In order to ensure that the U-Net reliably detects the pupil and not the iris, the synthetic data pipeline used for training the U-Net contained several more features than those for the pupil CNN. Firstly, a bright background representing the sclera with luminance $L_s \leftarrow \mathcal {N}(217,\,26)$ was generated. On top of this an iris was generated as a randomly positioned and oriented ellipse ($\alpha _i \in [30, 42.5]$ and major axis radius $\beta _i \in [1\alpha _i,1.3\alpha _i]$ and $L_i \leftarrow \mathcal {N}(77,\,16)$) rendered with an edge modulated by a raised cosine function over a range of between [8, 20] pixels. Then an irregularly shaped collarette was generated close to the center of the iris consisting of between 13 and 24 vertices ($N_{vert_{col}}$) arranged around the collarette center at an average distance $r_{col} \in [.3\beta _i,.6\beta _i]$, with the individual distance of vertices varied between $[0.05r_{col},0.2r_{col}]$. The resulting polygon was upsampled to five times the number of vertices using periodic cubic spline interpolation to create a shape with a smoothly varying edge, and the resulting polygon was rendered at luminance $L_{col}=[1.25L_i,1.6L_i]$ with an edge modulated by a raised cosine function over a range of between [1, 4] pixels.

On top of this were layered a randomly positioned and oriented pupil (minor axis radius $\alpha _p \in [10, 30]$ and major axis radius $\beta _p \in [1\alpha _p,1.3\alpha _i]$, $A_p \in [2,2000]$ and $L_p \leftarrow \mathcal {N}(34,\,15)$) and between one and eight ($N_{CR}$) randomly positioned and oriented CRs (minor axis radius $\alpha _c \in [0.8,4]$ and major axis radius $\beta _c \in [1\alpha _c,1.4\alpha _c]$, $A_c \in [2,20000]$ and $L_{CR}=255$), again avoiding overlap. The standard deviation of image noise was varied per generated image, with $\sigma _n \in [0,15]$.

#### Synthetic data pipeline for Chugh et al. (2021)

We use a synthetic data pipeline that improves on previous work (Maquiling et al., 2023) to perform pupil and CR localization and CR identity matching. The pupil is represented by a randomly oriented dark 2D Gaussian with a minor axis radius $\alpha _p \in [6, 22.5]$ pixels, major axis radius $\beta _p \in [1\alpha _p, 1.3\alpha _p]$ and amplitude $A_p \in [200, 100000]$. Its luminance $L_p$ is drawn from an exponential distribution with a scale parameter of 10 and offset 1.

Five randomly oriented CRs are generated, each having a random minor axis $\alpha _c \in [1, 2.5]$ pixels, a random major axis $\beta _c \in [\alpha _p, 1.1\alpha _p]$, and a random amplitude $A_c \in [200, 100000]$. Each CR has a drop-out rate *p* of 16%. Between one and five spurious (non-CR) reflections ($N_{spur}$) may randomly appear in the image. These are generated in the same way as CRs, each with a random minor axis radius $\alpha _s \in [1, 2.5]$ pixels and random major axis radius $\beta _s \in [\alpha _s, 2.5\alpha _s]$. The location of each spurious reflection is generated using a rejection sampling method with an inverted Gaussian ($1 - G(x, y)_p$, c.f. Eq 1) to make them less likely to appear near the pupil center.

A grayscale gradient background was created by drawing two random values from a luminance range of $L_i \in [63,178]$ and smoothly varying the luminance from one side to the other along a random axis. This is to prevent the model from interpreting any dark part of the image as part of the pupil. The standard deviation of image noise was varied per generated image, with $\sigma _n \in [0,30]$.

As this model not only performs pupil and CR center localization but also matching of CRs to specific illuminators, the positions of the CRs need to follow the same pattern as in the real dataset. Specifically, for the Chugh et al. (2021) dataset, this involves five IR lights that project to a house-shaped polygon that is usually close to the pupil. The polygon is modeled as a rectangle with an additional vertex above the middle of its top edge. The rectangle’s width is randomly sampled uniformly from $w \in [0.1d, 0.45d]$ where $d = 128$ pixels, the length of one side of the synthetic image. The separation between the two bottom vertices is then scaled by a factor $f_b$ uniformly sampled from [0.05, 0.2]. The rectangle’s height *h* is sampled uniformly from [0.5*w*, 0.6*w*], and the height of the roof $h_r$ uniformly from [0.2*w*, 0.5*w*]. The polygon is randomly rotated between $\phi =\pm [0, 45]$ degrees. In order for the model to learn the matching correctly, the CR positions are always calculated in a certain order, starting from the topmost position and moving clockwise.

In the second training stage, the maximum number of spurious reflections $N_{spur}$ that could appear in the image is reduced to 3, the dropout probability *p* for individual CRs is reduced to 10% and the range of rotation is reduced to $\phi =\pm [0, 35]$.

#### Synthetic data pipeline for OpenEDS 2020

We adjusted the synthetic data pipeline created for the Chugh et al. (2021) dataset, creating a polygon that has eight vertices corresponding to the eight IR lights in the dataset, starting from the bottom-right CR and moving clockwise. The polygon’s radius is randomly sampled uniformly from the range $m \in [0.15d, 0.4d]$ where $d = 128$ pixels. As the OpenEDS 2020 dataset contained forward-facing eye images, the random rotation of the polygon is reduced to the range $\pm [0, 0.57]$ degrees. Each CR has a dropout probability of 20%. The pupil luminance $L_p$ is drawn from a Weibull distribution with a scale of 25, an offset of 18 and shape parameter of 2, while no other parameters were changed.

### Real image pre-processing and output post-processing for model testing

As part of model inference, pre-processing of the input real eye images and post-processing of the model output was performed. Here we describe these routines for each model. It should be noted that these routines were developed specifically for the use cases and datasets to which our approach was applied. As such, an interested reader who wishes to apply the LEyes approach to a different problem domain would likely have to develop their own pre- and post-processing routines. However, since we cover a significant breath of eye-tracking related tasks with the models presented in this paper, we anticipate that readers interested in using LEyes can likely build on the pre- and post-processing routines we present here instead of having to develop their own routines completely from scratch.

Model inference was run on a laptop with a Xeon W-10885M CPU @ 2.40 GHz and an Nvidia Quadro RTX 3000 laptop GPU. The CNN models used for the high-resolution datasets ran on the CPU. Inference performance for the whole pipeline (including video decoding and the image processing described in this section) was 23 fps for the 500-Hz dataset and 27 fps for the 1000-Hz dataset. Inference using the three U-Net models for the VR datasets ran on the GPU at 37 fps for the EDS2019 dataset, 141 fps for the Chugh et al. (2021) dataset and 45 fps for the EDS2020 dataset.

#### High-resolution datasets

For the videos of the high-resolution datasets, image analysis was performed frame-wise using OpenCV (version 4.7.0.68) and the same steps as described in Byrne et al. (2023). Briefly, fixed pupil and CR thresholds were used to binarize the input images. The analysis was performed at different pupil and CR thresholds for each participant, and the thresholds that resulted in the best precision pupil and CR signals were used. After morphological operations to fill holes in the resulting blobs, size, shape, and relative location criteria were used to select the pupil and CR. The center of the pupil and the CR were then computed as the center of mass of these binary blobs. These centers will be referred to as the pupil and CR locations determined by the thresholding method.

In a second stage, 180$\times$180 pixel cutouts were made around the CR and pupil centers identified by the thresholding method. Similar to Byrne et al. (2023), a black circular mask with a radius of 32 pixels was applied to the CR cutouts. For the pupil, an ellipse was fit to the binarized pupil image from the thresholding method, and a middle gray elliptical mask was applied to the cutouts that was 1.4 times larger than the fitted ellipse. These masked CR and pupil images were the input for their respective CNNs.

#### OpenEDS 2019 dataset

For the OpenEDS 2019 dataset, the input images were downscaled by a factor of two after which a 192$\times$192-pixel cutout was taken from the center of the image. The segmentation output of the U-Net on this image, which is a probability map within the range of [0, 1], was first binarized using a threshold of 0.99. Subsequent post-processing then first refined the binary mask using morphological operations to eliminate any holes, after which the pupil was identified based on predefined shape and size criteria as was done for the high-resolution dataset above. The center of the pupil was then computed as the center of mass of the binary mask. An ellipse is furthermore fit to the binarized mask. To ensure that the pupil was fully contained in the initial centered cutout that was used as input to the U-Net, it was examined whether the identified pupil center was too close to the edge of the cutout. If the pupil center was estimated to be closer than the radius of the ellipse’s major axis from the edge of the cutout, the cutout was re-centered around the estimated pupil center, and the model’s inference was re-applied.

**Fig. 3:**
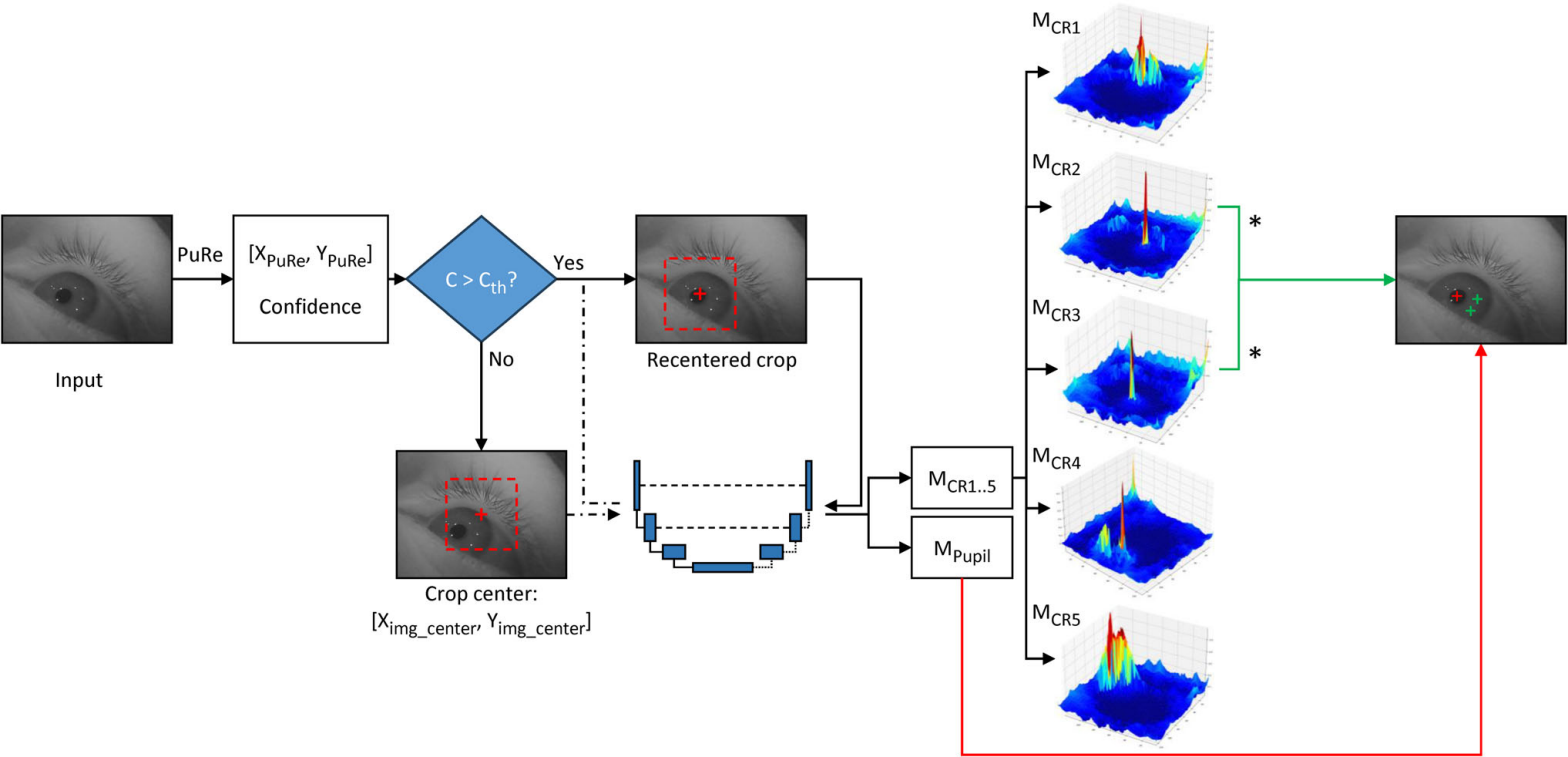
Flowchart of the simultaneous P-CR pipeline. First, an appropriate crop of the input image needs to be performed to provide input to the model. To locate this crop, an adaptive cropping strategy is used as follows. The center of the crop is determined using PuRe’s pupil center prediction ($[X_{PuRe}, Y_{PuRe}]$) if the confidence metric for PuRe’s prediction (*C*) is above a given confidence threshold ($C_{th}$), otherwise, the crop is determined by the pupil prediction of the LEyes-trained model given a naive center crop ($[X_{img\_center}, Y_{img\_center}]$). The pupil-centered crop is then passed through the model, which outputs logits representing likely feature locations for each prediction, illustrated here as heat maps (*M*) for both the pupil ($M_{Pupil}$) and for each CR ($M_{CR1...5}$ in this example). For each CR map and the pupil map, the highest value is located. These peaks are compared between CR maps and the two highest values across all the maps determine which CRs are selected. The *asterisks* signify which maps contain the two highest values in this example. However, if the exclusion criteria are met, the image is deemed invalid (see text)

#### Chugh et al. (2021) and EDS 2020 datasets

The models for the Chugh et al. (2021) and EDS 2020 datasets have an input resolution of 128$\times$128 pixels. Like above, the input images were downscaled by a factor of two after which a cutout was taken. The cutout was positioned as follows. A naive cutout strategy as used for the EDS 2019 dataset assumes that the pupil is close to the center of the eye image. Errors arise if the cutout excludes parts of, or even the entire, pupil. For these final datasets we developed a new method for solving this challenge. We employ PuRe (Santini et al., 2018a), a well-known lightweight open-source pupil detection method based on ellipse fitting, to create cutouts centered on the center of its detected pupil. Since in some cases PuRe fails to locate the pupil, we adopted an adaptive cutout strategy using PuRe’s confidence metric. This confidence, ranging between 0 and 1, with 0 indicating a poor estimated ellipse outline, is based on various metrics outlined in detail in Santini et al. (2018a). In our cutout method, if PuRe’s confidence is larger than or equal to a threshold (we used 0.70 for the Chugh et al. (2021) dataset and 0.90 for EDS 2020 as these delivered the best performance), the cutout used as input to the LEyes U-Net is based on PuRe’s pupil center estimate. If the confidence is below this threshold, we instead use a naive centered cutout and, similar to the strategy for the EDS 2019 dataset, take a second cutout centered on the detected pupil center of the first inference pass.

The LEyes U-Net model produces output maps for each feature (the pupil and each CR) that correspond to the confidence the model has that a given feature’s center is located at a given position in the input image. We will represent these unnormalized output values, which we will refer to as logit values, in the form of heatmaps (Fig. 3). The maximum value of each heatmap indicates the pixel location where the model is most confident that a certain feature’s center is located. While five (Chugh et al., 2021), or even nine (EDS 2020), CRs are expected in the image, for some eye positions some CRs will not be visible while at the same time spurious reflections may occur. Since robust eye tracking requires reliably associating at least two corneal reflections with their specific light source across all anticipated eye movements (Chugh et al., 2021), we have developed a method to robustly select only the ‘best’ two localized CRs based on the model output. To select the two CRs the model is most confident about, we choose the two CRs with the highest corresponding logit values across the output heatmaps. Selecting the ‘best’ two CRs is done independently for each frame. As such, which of the five (Chugh et al., 2021) or nine (EDS2020) CRs are selected may vary between frames. It should be noted that the model’s logit values reflect its joint confidence regarding CR center location and CR identity. Selecting by highest logit values thus optimizes on minimal CR identity confusion and localization errors, since such errors would propagate through a gaze estimation pipeline as large errors in gaze direction. Whether frame-to-frame changes in selected CRs are problematic depends on the gaze estimation pipeline and is outside the remit of this work. Should stable CR selection be required for an application, small modifications could be made to our pipeline to support, for instance, tracking selected CRs across frames as long as the model’s confidence remains above a certain threshold. To exclude eye images clearly unsuitable for eye tracking, for instance images that contain a blink or when both cutout strategies failed to capture the pupil, our method excludes images that fail to produce at least two CR heatmaps where the max values are greater than or equal to one.

### Data analysis for model testing

Model performance was evaluated as follows.

#### High-resolution datasets

Since the high-resolution datasets do not have a ground truth, a comparative analysis of data quality comparing standard thresholding methods to the LEyes dual CNN method was performed.

The data quality of the eye-tracking signals for both high-resolution datasets was determined using the same method as Byrne et al. (2023). Briefly, RMS-S2S precision (Holmqvist et al., 2012; Niehorster et al., 2020a, b) of the pupil and CR center location signals estimated by both the thresholding and LEyes CNN methods was computed in a 200ms window moved over the signals, after which each trial and signal’s median RMS values were determined (Hooge et al., 2022, 2018; Niehorster et al., 2020).

We computed calibrated gaze signals by subtracting the CR center location from the pupil center location for the thresholding, LEyes CNN and EyeLink data, and calibrating the resulting vector with data from the 3x3 grid of fixation points from the first task. We used second-order polynomials in *x* and *y* with first-order interaction terms to calibrate the P–CR gaze signal (Cerrolaza et al., 2012; Stampe, 1993). To examine the quality of the resulting calibrated gaze data, we computed accuracy as the offset between the estimated gaze location and the target location for the gaze data from task six, which involved repeated fixations on 15 targets. We determined the RMS-S2S precision of the calibrated gaze signals for all recorded trials in the same way as for the pupil and CR center signals, and computed the standard deviation of the signals using the same sliding window technique.

**Fig. 4:**
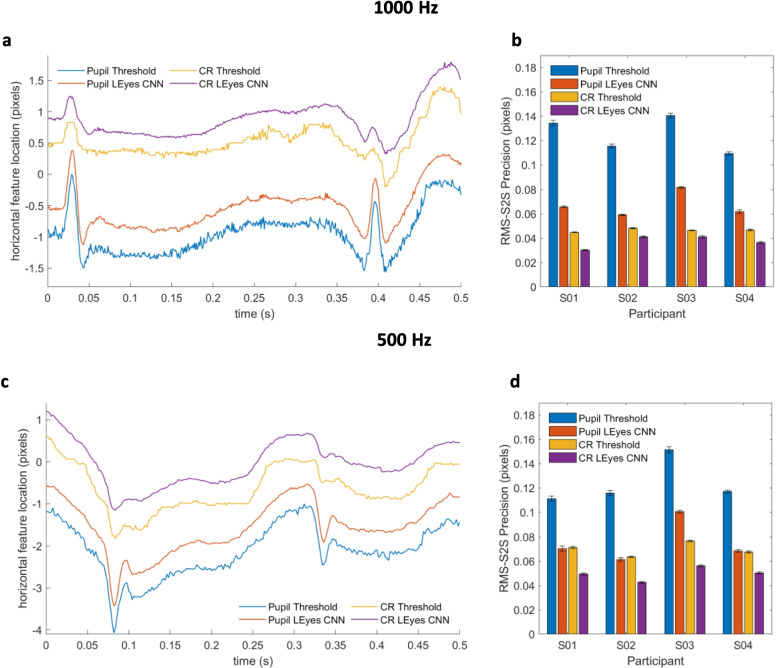
CR and pupil center signals for the high-resolution datasets. *Left column*: representative segment of raw pupil and CR center signals derived from eye images recorded at 1000 Hz (**a**) and 500 Hz (**c**). The two CR signals and the two pupil signals in both panels have been vertically offset from each other (0.4° in panel (**a**), 0.6° in panel (**c**)) for clarity. Right column (panels **b** and **d**): an RMS-S2S precision comparison between the thresholding and LEyes CNN methods for the pupil and CR signals on all data of four participants. *Error bars* depict standard error of the mean

#### OpenEDS 2019 dataset

For all the VR datasets, pupil center localization accuracy was assessed using the ground truth provided in the datasets as standard. Center of mass computations were used to determine the pupil center location from the pupil segmentation annotations provided in the OpenEDS 2019 (Garbin et al., 2020) and OpenEDS 2020 (Palmero et al., 2021) datasets. Since input images were downscaled to the model’s input size, the determined pupil center location was scaled back to the original image size to allow for fair comparison.

In the literature, feature localization accuracy is often evaluated using the cumulative detection rate, which shows how many of the pupil locations estimated by a method are within a given distance from the ground truth pupil center (e.g., Fuhl et al., 2017; Kim et al., 2019; Kothari et al., 2021; Santini et al., 2018a). Performance is often furthermore specifically assessed as the percentage of images for which the pupil location was estimated within five pixels from the ground truth (Kim et al., 2019; Fuhl et al., 2017). We adopt these measures to evaluate the performance of the LEyes U-Net on the EDS 2019 dataset. However, recent algorithms have demonstrated superior performance and often reach ceiling performance well below this five-pixel threshold. Consequently, we have narrowed our analysis to examine performance for errors up to just two pixels.

Using these metrics, we compared our results with other state-of-the-art models and frameworks, including Pistol (Fuhl et al., 2023), PuRe (Santini et al., 2018a), the EllSeg framework (Kothari et al., 2021), and DeepVog (Yiu et al., 2019). These models employ a variety of methods, ranging from conventional ellipse fitting to deep learning architectures. Note that we stress the difference between model and framework where a “model” is a specific representation trained to make predictions, while a “framework” is a set of tools and libraries used to develop, train, and deploy such models.

**Fig. 5:**
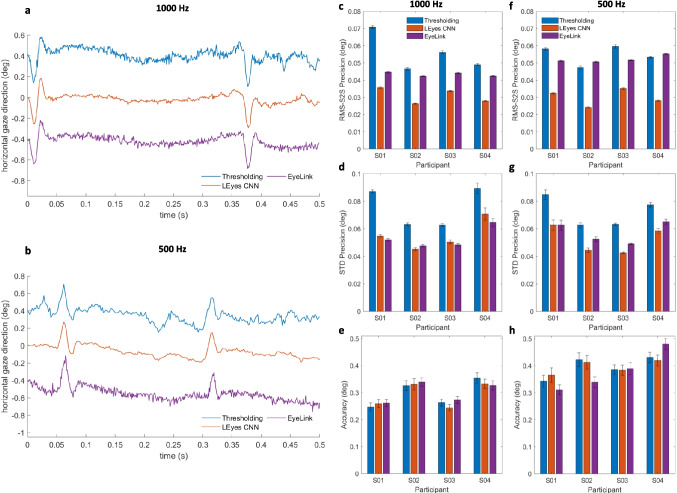
Gaze signals calibrated using a polynomial (see the “High-resolution datasets” subsection in the “Data analysis for model testing” section above). *Left column*: representative segment of calibrated P-CR signals derived from 1000-Hz data (**a**) and 500-Hz data (**b**) as derived from pupil and CR center locations determined using either thresholding or the dual LEyes CNN approach, along with the EyeLink. The signals in both panels contain two small saccades and have been vertically offset by 0.4° for clarity. Further, RMS-S2S precision, STD precision, and accuracy comparisons for the 1000-Hz data (*middle column*, panels **c**–**e**) and the 500-Hz data (*right column*, panels **f**–**h**) between the three gaze-tracking methods on data of all participants are shown. *Error bars *depict standard error of the mean

#### Chugh et al. (2021) and EDS 2020 datasets

For the Chugh et al. (2021) and EDS 2020 datasets, mean pixel error (upscaled to undo image downscaling to the models’ input size) was used to evaluate pupil center localization performance. Furthermore, for the Chugh et al. (2021) dataset, which also provides CR center annotations, correct CR detection rate (error less than five pixels, as adopted by Chugh et al., 2021) and the mean pixel error for correctly detected CRs was also assessed.

## Results

### Dual CNN P-CR eye tracking

The Pupil-Corneal Reflection (P-CR) eye-tracking method, often employed in controlled lab settings for gaze estimation, requires accurate identification of both the pupil and Corneal Reflections (CRs) (Hooge et al., 2016; Nyström et al., 2023; Fuhl et al., 2017). When estimating the smallest and fastest of eye movements, an eye tracker with high spatial and temporal resolution is required. This typically requires sub-pixel localization of the pupil and CR(s). Researchers using eye tracking are rarely interested in the individual pupil and CR signals, but instead use the gaze signal derived from them.

Here, we examine whether the LEyes CNNs deliver improved pupil and CR center signals compared to standard thresholding methods, and whether the improved precision of the pupil and CR center signals lead to an improved gaze signal. We furthermore compare the gaze signal of our method to that delivered by the SR Research EyeLink 1000 Plus.

Example raw pupil and CR signals resulting from thresholding and the LEyes CNNs are shown in Fig. 4a (1000 Hz) and 4c (500 Hz). As can be readily appreciated, the sample-to-sample variation in both the pupil center and the CR center signal is lower for the LEyes method than for the standard thresholding method for data acquired at both sampling rates. To formalize this observation, the precision in the form of root mean square of sample-to-sample deviations in the signal (RMS-S2S) was computed across the datasets and plotted in Fig. 4b (1000 Hz) and 4d (500 Hz). This analysis confirms that for both the 1000- and the 500-Hz data sets, the LEyes CNNs consistently demonstrated superior precision (lower values) than the thresholding method.

Do the improved pupil and CR center signals translate to an improved gaze signal? This was examined using calibrated P-CR gaze signals computed from pupil and CR centers estimated by thresholding or by the LEyes CNNs and both were compared with the gaze signal delivered by the EyeLink. Example gaze signals are plotted in Fig. 5a (1000 Hz) and 5b (500 Hz). Again, it can be readily appreciated that the gaze signal derived from the LEyes CNNs is smoother and more stable than that derived from standard thresholding operations, but also than that delivered by the EyeLink. To quantify this observation, we computed the RMS-S2S precision of these signals, which quantifies short-timescale smoothness, as well as the STD precision (Niehorster et al., 2020b), which quantifies the spatial spread of the signal and indicates its stability. These evaluations are presented in Fig. 5c–d (1000 Hz) and 5f–g (500 Hz). This analysis confirms that the dual LEyes CNN method consistently demonstrated superior RMS-S2S precision (lower values) than the thresholding method and the results from the EyeLink 1000 Plus. It is important to note that both of our methods processed each video frame independently, without using any temporal information from preceding or future frames. Thus, the increased precision seen in the CNN method cannot be attributed to the use of temporal information (Niehorster et al., 2021, 2020b). The signal stability (STD precision) achieved by the LEyes method was on par with the EyeLink for the 1000-Hz dataset and slightly better than the EyeLink for the 500-Hz dataset, and consistently outperformed the thresholding method for both datasets. The achieved accuracy (Fig. 5e and h) did not systematically differ between the three methods, indicating that the gains in precision did not come at the cost of reduced accuracy.

**Fig. 6:**
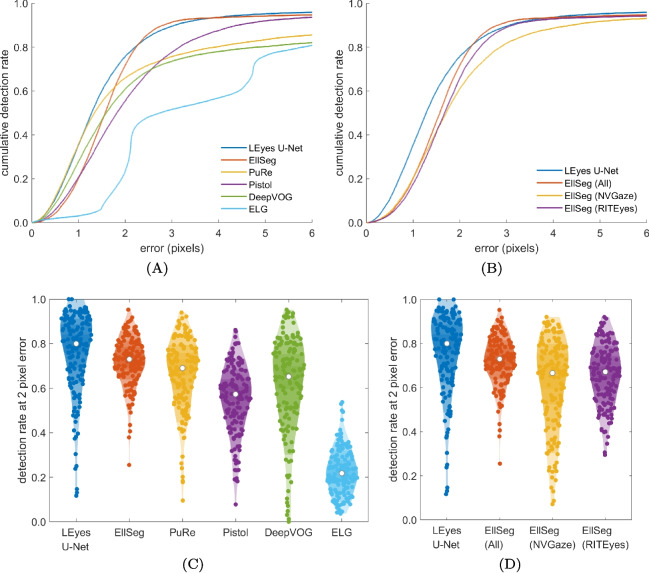
**A.** The cumulative detection rate on the OpenEDS 2019 dataset of a U-Net model trained using the LEyes method at different pixel errors compared against PuRe (Santini et al., 2018a), Pistol (Fuhl et al., 2023), DeepVOG (Yiu et al., 2019) and ELG (Park et al., 2018). **B.** Comparison of the LEyes U-Net with several models trained using the EllSeg Framework (Kothari et al., 2021, 2022). **C. & D.** The corresponding violin plots for panels A. and B., respectively, showing the detection rate at an error of 2 pixels for each participant in the testing set, comparing the LEyes U-Net with the aforementioned models. The *white dots* in these figures indicate the median

### Pupil localization

Next, we consider the performance of the LEyes framework in a pupil center localization task in a VR setting using the OpenEDS 2019 dataset (Garbin et al., 2020). We chose this dataset to run a comparative analysis as it has been used extensively to assess the accuracy of other methods in gaze estimation tasks (Chaudhary et al., 2022; Kim et al., 2019; Kothari et al., 2022; Nair et al., 2020). We compare our results with other state-of-the-art models and frameworks; Pistol (Fuhl et al., 2023), PuRe (Santini et al., 2018a), the EllSeg framework (Kothari et al., 2021), DeepVog (Yiu et al., 2019), and the model by Park et al. (2018).

As illustrated in Fig. 6, we achieved a two-pixel error rate of 75.8% (indicating that the localization error was below 2 pixels for 75.8% of all images in the EDS2019 set), which is close to EllSeg (model trained on all datasets) at 71.8% and surpasses Pure (65.6%), DeepVOG (60.9%), and Pistol (55.4%). The violin plots in the bottom section of Fig. 6 split out these results per participant. Each data point in this plot is the two-pixel error rate for one participant in the EDS2019 dataset. This comparison shows that on the participant level, LEyes achieved a median two-pixel error rate of 80%, outperforming the next-best model by 7%.

We underscore comparisons with the different variants of the EllSeg framework (Kothari et al., 2021), one of the few other public frameworks leveraging synthetic training data. The orange line in Fig. 6(B) shows the EllSeg variant trained across multiple eye datasets. Notably, OpenEDS 2019 is one of the datasets included in its training set. Astonishingly, our LEyes model still performs on par with this variant, even though there is evident data leakage in the way we evaluated this EllSeg variant since 88.6% of the test set we used was present among its training samples. Two further comparisons of EllSeg models trained on purely synthetic datasets (RITeyes, Nair et al., 2020; and NVGaze, Kim et al., 2019) show that the LEyes framework consistently outperforms other publicly available models that use only synthetic data.

Taken together, the results highlight that LEyes achieves performance that is on par with or higher than other methods tested on the EDS 2019 dataset. In line with earlier observations on domain discrepancies and generalization in gaze estimation (Kothari et al., 2022; Nair et al., 2020), our results demonstrate that models exhibit optimal performance when trained on datasets analogous to their respective test distributions, something that is easily achieved within the LEyes framework.

### Simultaneous pupil and corneal reflection localization

Pupil and CR localization can be treated as two separate problems, yet since the positions of these features co-vary in the eye images in a systematic way, it may be advantageous to consider them together. In this section, we present a new P-CR eye-tracking pipeline that simultaneously localizes pupil and CR centers, trained entirely using LEyes images. Eye trackers often use several light sources to guarantee at least a pair of reflections for every gaze position, and for robust eye tracking it is necessary to reliably associate at least two corneal reflections with their specific light source across all anticipated eye movements (Chugh et al., 2021). Therefore, our pipeline is not only able to localize the pupil and CR centers in an eye image, but also match the CRs to specific light sources.

While previous work has developed models that locate the pupil and CRs simultaneously and perform CR matching (Niu et al., 2021; Maquiling et al., 2023), we introduce a novel method that streamlines the process of robust P-CR eye tracking by using the maximum value of the model output to select only the ‘best’ two CRs. This ability of our method to robustly select CRs for gaze estimation is especially important due to the complex reality often encountered in eye images where CRs may be missing and additional, unwanted, reflections are often present.

**Fig. 7:**
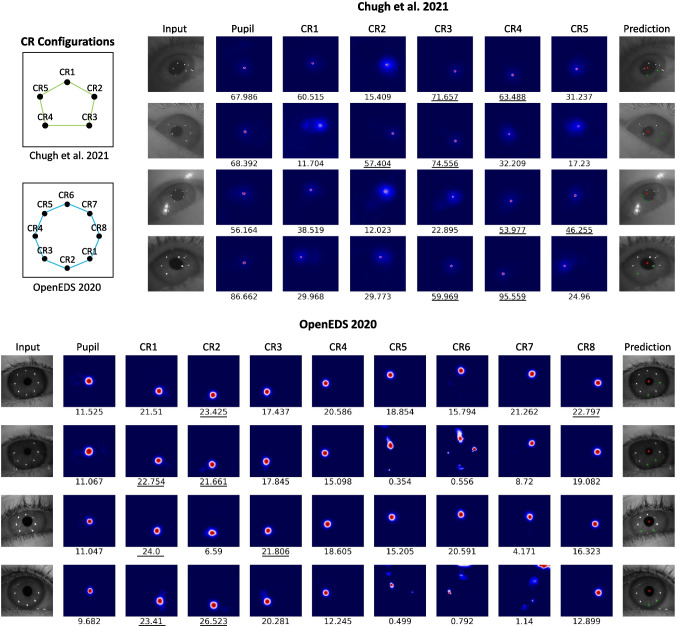
Heat maps showing output of our models for both the Chugh et al. (2021) dataset and the OpenEDS 2020 dataset. The maximum corresponding logit value (corresponding to the model’s confidence in the localization, higher is better) is shown under each heat map. In the Chugh et al. (2021) dataset, the labeling of the CRs starts at the top-most IR reflection and then proceeds clockwise (*top right*). In the OpenEDS 2020 dataset, the labels used when training the model start at the lower right CR and proceed clockwise. Our algorithm selects the two highest logit values from the CR maps (*underlined*) along with the pupil value for a complete robust P-CR pipeline. The *last column* shows the predicted locations of the centers of the pupil and selected CRs on the corresponding eye image

**Fig. 8:**
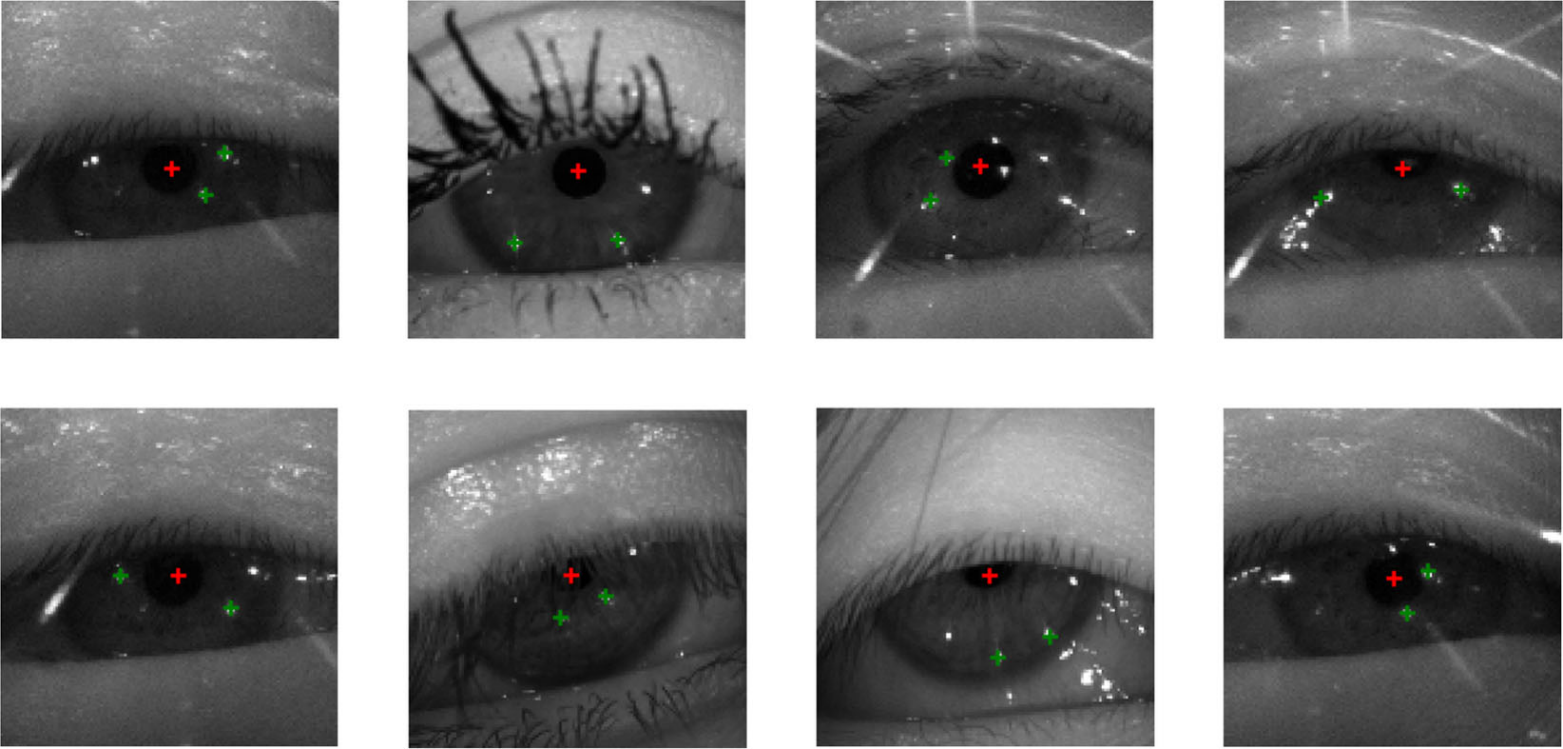
Predicted locations of the centers of the pupil (*red cross*) and selected CRs (*green crosses*) on eye images from the EDS2020 dataset. Shown are various more challenging images (partially occluded pupils, spurious reflections)

As described in the method section and outlined in Fig. 3, our image processing pipeline first runs PuRe (Santini et al., 2018a) on the input eye images. We found that PuRe by itself had average errors of 13.2 and 20.8 pixels in the OpenEDS 2020 and Chugh et al. (2021) datasets, respectively. The next step is to provide a cutout centered on the center of PuRe’s pupil estimate to the LEyes model. This still yielded high average pupil center errors of 11.0 and 7.76 pixels in those datasets due to cases where PuRe failed to locate the pupil. As detailed in the methods section, this was mitigated by only using PuRe’s estimate of the pupil center to position the cutout that is fed to the LEyes model if PuRe’s confidence is above a certain threshold value. The red crosses in the right columns of Fig. 7 show example pupil center localizations using this strategy for both datasets. Using this strategy, for the OpenEDS 2020 dataset, the lowest average pupil error was 4.18 pixels, achieved when using a confidence threshold of 0.90 (the largest average error was 4.76 pixels for confidence thresholds between 0.50 and 0.95). For the Chugh et al. (2021) dataset, an average error of 4.15 pixels was achieved at the 0.70 confidence threshold (largest error for confidence thresholds between 0.50 and 0.95 was 5.11 pixels).

Besides pupil centers, our model simultaneously localizes CRs and identifies the corresponding illumination source for each CR. Figure 7 shows the heat maps of each CR and their associated max values derived from a few example real eye images for both the Chugh et al. (2021) and the OpenEDS 2020 datasets. Furthermore, the predicted locations of the two ‘best‘ CRs are shown as green crosses in the rightmost columns of the figure. To assess the performance of our model on CRs, we compared the correct CR detection rate to that achieved by the model of Chugh et al. (2021) and the determined the error in CR localization using the CR annotations in the Chugh et al. (2021) dataset as reference. Chugh et al. (2021) achieved successful matches of at least two CRs within five pixels for 91% of the images in their test set and an average error of 1.5 pixels on these images. It is worth mentioning that Chugh et al. (2021) had to sacrifice 88% of the dataset for both training and validation of the model, so their results include only a small part (12%) of the whole dataset.

In contrast, since LEyes is trained on synthetic images, we can evaluate our model on the entire dataset. Therefore, direct comparisons between the two models are not straightforward since they are evaluated on a different number of images and use different exclusion criteria. To make the results more comparable, we apply our exclusion criterion that the maximum value of at least two heat maps is larger than 1 in conjunction with the criterion of Chugh et al. (2021) that evaluates model performance only on the images where the predicted locations of at least two CRs were less than five pixels away from the ground truth. Using these criteria, our model exhibited an average pixel error of 1.59 across all the CRs. Focusing solely on the two CRs selected by our method (highest activation in the probability map output by the network), this error was reduced by 18% to 1.30 pixels. These errors are in line with those reported by Niu et al. (2021) on their own non-available dataset of images that included four CRs. Further, using both exclusion criteria we retain 70% of images from the dataset.

Since only pupil, but no CR, annotations are provided with the OpenEDS 2020 dataset (Palmero et al., 2021), we provide illustrative examples instead of a quantitative comparison. Figure 7 (bottom) illustrates how our model performs on a representative selection of eye images from the OpenEDS 2020 dataset, and Fig. 8 shows performance on some more difficult images. Predictions from all eye images in the dataset are available in the repository associated with this paper. Through these examples, we want to highlight that our model appears to provide accurate predictions also for CRs in this dataset, despite having more CRs (eight instead of five) with a different spatial configuration compared to the Chugh et al. (2021) dataset.

## Discussion

In this paper, we presented LEyes, a novel framework for training gaze estimation algorithms using synthetic data. The main difference between LEyes and previous methods and frameworks for training gaze estimation models on synthetic data (Nair et al., 2020; Chaudhary et al., 2022; Kim et al., 2019; Kothari et al., 2021) is that while previous methods use photorealistic rendering of eye images for training, LEyes uses abstract representations of eye images that to a human observer do not look like the near eye images an eye tracker would capture. Despite this simplification, which allowed us to generate training data on the fly and train our deep learning models in a computationally efficient manner, LEyes achieved cutting-edge results for both high-resolution, lab-based eye-tracking and virtual reality eye-tracking setups.

In the high-resolution setting, LEyes exceeded the performance of the EyeLink 1000 Plus eye tracker across two lighting conditions in terms of noise level in the resulting gaze signals. In the VR setting, first, LEyes performed on par with or outperformed other methods in a pupil center localization task by a margin of at least 4%. Second, we introduced a novel LEyes-trained P-CR pipeline that both simplifies and improves CR detection by considering only the two best CRs in the recorded image, achieving results that are on par with previous work using models trained on part of the real image dataset used for testing (Chugh et al., 2021; Niu et al., 2021). Overall, our results emphasize both the accuracy and flexibility in design of the LEyes framework, highlighting its applicability across gaze estimation applications, and indicating that using highly simplified eye images for training deep learning models is a worthwhile avenue to explore in further research. Since the localization of pupil and CR centers comprises an initial step for both feature-based and model-based eye-tracking approaches (Hansen & Ji, 2010; Liu et al., 2022), LEyes could be used for both.

LEyes significantly reduces the amount of data required to conduct an eye-tracking study that uses a deep learning model to analyze the data, by eliminating the need to sacrifice recorded data for model training and validation, resulting in both time- and cost-savings. For example, our model was able to run inference on the entirety of the Chugh et al. (2021) dataset, while the original paper used 88% of the data for both training and validation and were thus left with only 12% for evaluating their model (Chugh et al., 2021; Maquiling et al., 2023). Furthermore, LEyes offers an alternative to the challenging task of creating photorealistic synthetic data. Many researchers may not possess the skills, time, or resources to access and use software platforms like Blender or Unity3D. Finally, when combined with the FLEX system (Nyström et al., 2023; Hooge et al., 2021) which has a hardware cost of about $1000 USD or other open eye-tracking platforms (see Niehorster et al., 2025a, for an overview), LEyes may offer a promising low-cost and open-source alternative to eye trackers such as the SR Research EyeLink 1000 Plus.

Although we think that our work offers a promising new direction for deep learning models for eye tracking, the LEyes approach has several open problems that offer compelling avenues for future research. Furthermore, there are trade-offs between using LEyes and the traditional real image and photorealistic synthetic image approaches.

First, as discussed in the methods section, building a LEyes image synthesis pipeline for a given dataset requires both knowledge about the physics of the problem (such as appropriate shapes of the eye features in the image and relative luminance relations between the features) and examination of the luminance distributions of features in the testing dataset. As such, part of setting up the LEyes pipeline for a specific dataset was to determine the mean luminance of the pupils and of the region around the pupil (part of the iris) and their standard deviation in the dataset. These luminance distributions were used to inform parameters in the LEyes synthetic image pipeline. Note that we did not use the exact empirical distributions from the real image datasets; we only used the distributions as guidance to ensure that the distributions used for LEyes images were not far outside those of the testing dataset. Nonetheless, this workflow requires that the testing dataset is first analyzed for its luminance distributions before the LEyes pipeline can be set up. Due to this, LEyes is not currently suitable for autonomous operation on new datasets. This stands in contrast to other models that have been designed to be trained once and then applied to a large number of use cases (e.g., Fuhl. et al., 2023; Kothari et al., 2021). That LEyes underwent some adaptation to each dataset may help explain why LEyes often outperforms the other approaches, although it should be noted that for instance EllSeg was trained on the EDS2019 dataset used for evaluation in this paper.

There are, however, multiple interesting avenues to explore to alleviate or entirely remove the limitation that manual work is required to adapt LEyes to a specific dataset. One direction to explore would be whether it is possible to use a LEyes synthetic image pipeline to train a more generic and powerful model that offers good performance for detecting target eye features across a range of different datasets. The creation of such a model would sidestep the problem of having to handcraft an appropriate LEyes synthetic data pipeline for a specific eye-tracking setup. A further direction is to explore how much of a dataset has to be analyzed to set the parameters for the synthetic training image pipeline. Given that the empirical luminance distributions of a dataset were only used as guidance when determining the parameters, we expect that hand-annotating only a few images from a few participants could be enough to retrieve the distributions typical for images recorded in a given setup and thus sufficient to tune the distributions that LEyes is trained on. This would all but remove this bottleneck, and allow constructing a LEyes model that can be used for all recordings made on a given setup based on a small pilot data collection. A final direction to explore would be to use vision foundation models such as SAM2 (Ravi et al., 2024), which requires minimal user interaction and no training data due to its strong performance in zero-shot segmentation tasks for eye images (Maquiling et al., 2024; Niehorster et al., 2025b). This model could be used to segment the pupil, iris and even the skin and other background features in the images when of interest, facilitating rapid estimation of the luminance distributions in the dataset which would enable automatically configuring the parameters for the LEyes synthetic image pipeline.

Second, gaze estimation models trained on synthetic data may struggle to adapt well to the application domain of a certain dataset with real images (Nguyen et al., 2024; Kim et al., 2019; Kothari et al., 2022; Eskildsen & Hansen, 2020). We therefore, despite the good performance that LEyes has demonstrated, think it worthwhile to explore to what extent domain adaptation techniques using real images can improve the performance of LEyes-trained models. Such work would explore the performance impact of applying domain adaptation techniques such as fine-tuning of models trained using synthetic images generated with LEyes using only a few annotated real eye images. Alternatively, the opposite scenario might also be considered, i.e., using LEyes images to generalize a model trained on limited real data or adapt such a model to the distribution of another dataset. Besides such domain adaptation techniques there is also the possibility of using a hybrid training dataset consisting of real data from only one participant augmented with LEyes images with appropriately matched distributions. All these approaches would allow to efficiently adapt models to a given eye-tracking setup or even a specific participant while sacrificing minimal data. While these approaches would require some real data to be collected and annotated, they nonetheless have the potential to significantly reduce the required resources to construct a powerful training dataset. Recent work on efficiently selecting training images to facilitate out-of-domain generalization (Biswas & Lescroart, 2024) should be explored as part of these efforts as they may further reduce the amount of real data needed when pursuing these approaches.

Third, during the process of training and evaluating the LEyes models, we found that not all models that trained well on the synthetic images also performed well on real images. Even though the synthetic images were tailored to the task at hand using knowledge about the physics of the problem (such as appropriate shapes of the eye features in the image and relative luminance relations between the features) and examinations of the luminance distributions of features in the testing dataset, a small amount of trial and error proved to be unavoidable in creating synthetic data to train models that performed well on real images. Such trial and error involved making small changes to the distributions of feature shapes, luminance and relative positions (e.g., making pupil-like shapes darker or lighter). The derivation of a loss function for training which maps to performance on real images would presumably alleviate this problem and remains as an important direction for future research.

Lastly, it should be noted that while the current paper pushes our earlier work (Byrne et al., 2023) using simple synthetic images to train a model for localizing the center of a CR to significantly more complex tasks (such as simultaneously localizing pupil and corneal reflection centers) and scenarios (lower-quality eye images for VR systems containing multiple CRs as well as spurious reflections), further examining the potential of our approach for feature detection in even more challenging datasets remains. Of particular interest would be targeting outdoor wearable eye-tracking datasets which are notorious for the sometimes very poor quality eye images that can pose significant challenges to any feature detection algorithm (e.g., Fuhl et al., 2016, 2021; Eivazi et al., 2019; Kim et al., 2019; Kothari et al., 2020).

In conclusion, prior to LEyes, the development of gaze estimation algorithms using machine learning was confined to those who possessed the resources to amass large annotated datasets or the technical expertise and large computational resources to generate synthetic data. We posit that with LEyes, the training of deep learning models for gaze estimation has become easily accessible to everyone, democratizing the field and opening new avenues for exploration and application.

## Data Availability

We do not have permission to share the high-resolution eye videos we have collected. The models employed in our experiments are available at https://github.com/dcnieho/Byrneetal_LEyes.

## Code Availability

The code used to generate LEyes synthetic data and to train the models employed in our experiments are available at https://github.com/dcnieho/Byrneetal_LEyes.

## Declarations

### Open Practices Statement

The experiments were not preregistered. We have made available the code used to generate LEyes synthetic data and the models employed in our experiments in a GitHub repository at https://github.com/dcnieho/Byrneetal_LEyes. We do not have permission to share the high-resolution eye videos we have collected.
